# Bilateral pulmonary sequestration in the elderly adult

**DOI:** 10.1186/2049-6958-7-36

**Published:** 2012-10-22

**Authors:** Ersin Sukru Erden, Hanifi Bayarogullari, Hatice Bilgic, Tulin Yetim, Eyup Buyukkaya

**Affiliations:** 1 Department of Chest Diseases, Faculty of Medicine, Mustafa Kemal University, 31000, Hatay, Turkey; 2 Department of Radiology, Faculty of Medicine, Mustafa Kemal University, Hatay, Turkey; 3 Department of Thoracic Surgery, Faculty of Medicine, Mustafa Kemal University, Hatay, Turkey; 4 Department of Cardiology, Faculty of Medicine, Mustafa Kemal University, Hatay, Turkey

**Keywords:** Congenital abnormality, Bronchopulmonary sequestration, Lung neoplasms, Humans, Female, Middle aged

## Abstract

Pulmonary sequestration (PS) is a rare malformation consisting of aberrant lung tissue which is not affiliated with the normal bronchial system and is fed by an aberrant artery that derives from systemic arteries. However, PS is usually seen unilaterally but, only rarely, it is bilateral. Most patients with PS are diagnosed because of symptoms due to pulmonary infection or cardiac disease, while a small portion of patients are asymptomatic and diagnosed incidentally. In this report, we present an extremely rare case of asymptomatic bilateral PS which was diagnosed at advanced age. To our knowledge, this case represents the oldest patient in the literature, and the second case that was diagnosed in a patient over the age of 50.

## Background

Pulmonary sequestration (PS) is a relatively rare anomaly and consists of a lung field which is not connected with the bronchial system. Arterial supply is derived from the aorta or aortic branches and venous drainage is usually to the left atrium via pulmonary veins [1]. PS is usually unilateral, and bilateral pulmonary sequestration is a very rare anomaly [2]. Here we present a case of asymptomatic bilateral pulmonary sequestration, which was diagnosed with pulmonary CT angiography at advanced age.

## Case presentation

A 56-year-old female patient suffering from dry cough, continuing after upper respiratory tract infection, was referred to pulmonary diseases clinic by the attending physician for the suspicion of malignancy because of abnormal findings on chest radiography, consisting in a left lung lower lobe mass with lobulated contour that included cystic areas, and a right lung lower lobe mass in the paravertebral region on thorax CT scan. The patient had no complaint except dry cough. Her medical history was unremarkable, there was no history of previous disease, chronic cough and sputum production, hemoptysis, chest pain, shortness of breath or recurrent pulmonary infections.

The physical examination revealed a BP of 110/60 mm Hg; pulse rate : 72 beats/min; respiration rate : 16 breaths/min; and body temperature : 36°C. Respiratory system examination revealed decreased breath sounds in left lower lung and a systolic murmur was heard in the medial part of the lower zone. Other physical examinations and laboratory findings, including complete blood count and C reactive protein, were normal.

**Figure 1:**
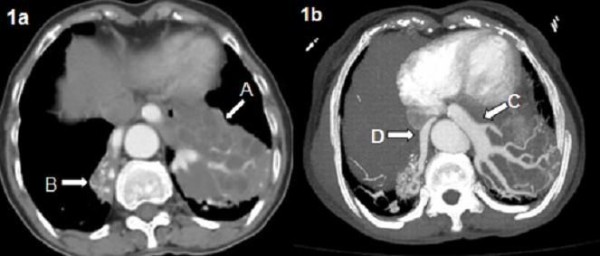
**(a) On chest CT transverse section aberrant lung tissue in the posterobasal left lung (*arrow A*) and posterobasal right lung (*arrow B*) are seen.** (**b**) Aberrant arteries are seen leading to aberrant tissue on the left (*arrow C*), and the right (*arrow D*).

**Figure 2:**
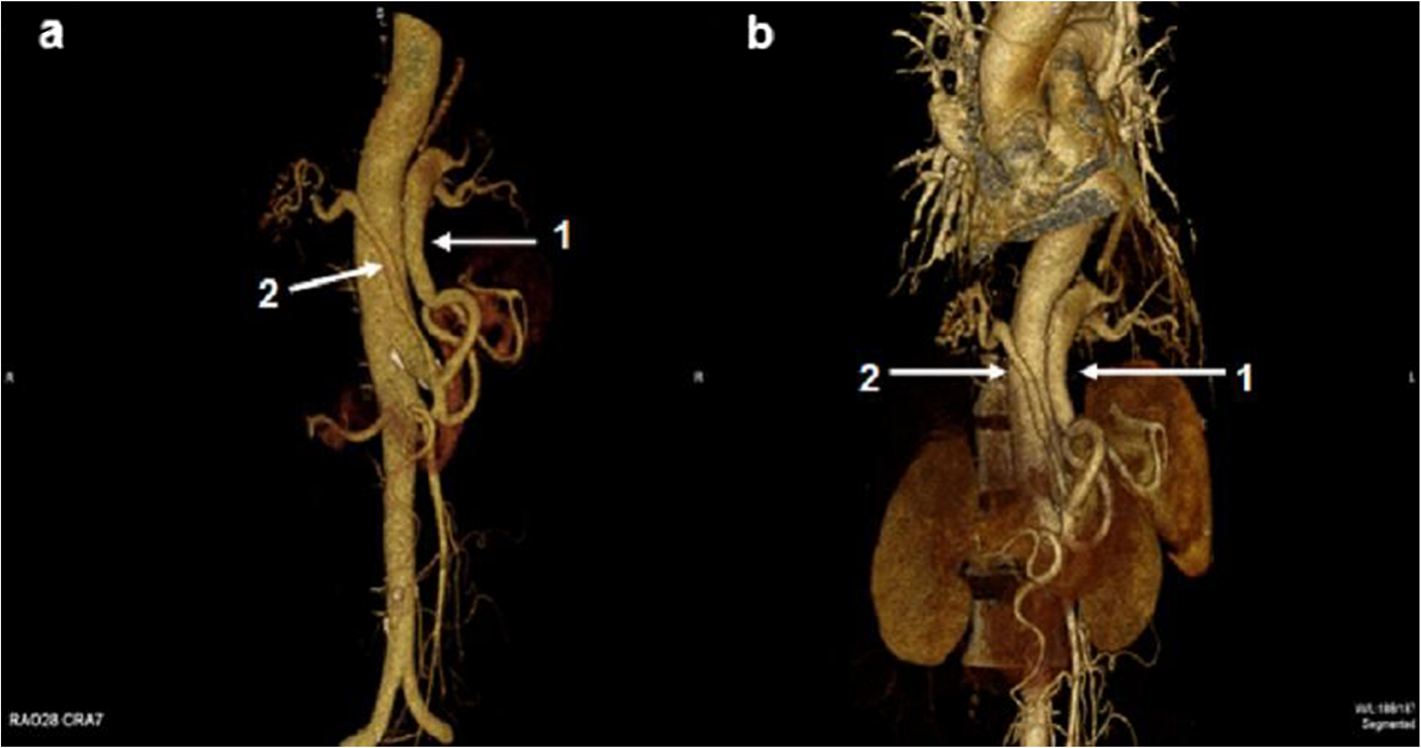
**(a, b) 3D volume rendered image shows the aberrant artery branched from celiac truncus and supplying the sequestration on the left (*arrow 1*) and the aberrant artery supplying the sequestration on the right (*arrow 2*).**

The patient's chest CT was evaluated by a radiologist, and the mass lesions in the CT scan were thought to be compatible with a PS, but that examination was considered insufficient to confirm the diagnosis. Then, chest CT angiography was performed. It showed abnormal tissue compatible with bilateral PS in the posterobasilar lower lobes bilaterally with heterogeneous internal structure, including cystic areas, and two aberrant feeding arteries branching from celiac truncus with venous drainage to the pulmonary veins (Figures 1, 2). The echocardiographic evaluation was normal. The patient was recommended surgical treatment for the bilateral intralobar PS, but she refused, so that a clinical follow up is ongoing.

## Discussion

Pulmonary sequestration is a bronchopulmonary mass lesion, with cystic or solid structure, separated from normal lung and is not functional [3]. Two types of sequestration are described: intralobar and extralobar. Sequestration located within the visceral pleura with normal lung tissue is referred to as intralobar. Sequestration, that is completely covered by a separate pleura, is called extralobar. The vast majority of sequestrations (75%) are intralobar [4]. Incidence of intralobar PS ranges from 0.15% to 1.7% and it constitutes 6.4% of all congenital pulmonary anomalies. It is most often observed in the posterobasal segment of the lower lobe [1]. A review study, in which Savic et al. evaluated 540 pulmonary sequestration cases, showed that there were only two bilateral intralobar sequestration cases [1]. Half of the patients with intralobar PS are diagnosed before the age of 20 [5]. Savic et al. reported that first symptoms occurred before the age of 10 in 37.2% of patients, 15.5% of the cases were asymptomatic and diagnosed incidentally [1].

Patients with PS are often diagnosed with symptoms due to secondary infection or cardiac disease. Recurrent pneumonia, chest pain, shortness of breath, hemoptysis, abscess, fever, fatigue and rarely clubbing of the fingers can develop. Cardiovascular findings may occur depending on the right-to-left or left-to-left shunts [6].

Stern et al. compiled a total of 17 patients with bilateral PS, including a case of neonate with bilateral intralobar PS. In this review, the mean age at diagnosis was 1.7 months in the 7 cases diagnosed early, whereas it was 16.1 years in the remaining cases. In addition, the most common presentation of the disease is recurrent respiratory infections [2]. Enfield et al. reported a 53-year-old asymptomatic bilateral PS case. An asymptomatic male patient with pulmonary mass on routine chest X-ray was diagnosed to be affected with bilateral PS at the CT angiography [7].

The differential diagnosis of PS includes lung abscess, lung cancer, bronchiectasis, congenital cystic adenomatoid malformation, empyema, congenital diaphragmatic hernia and pneumatocele due to staphylococcal pneumonia [8].

## Conclusions

The case presented here was referred to outpatient pulmonary diseases clinic by the attending physician for the suspicion of lung malignancy. The patient had neither symptoms like chronic cough and sputum production, hemoptysis, shortness of breath, orthopnea, paroxysmal nocturnal dyspnea, nor history of recurrent bronchitis or pneumonia. There was no evidence of heart failure on physical examination and echocardiographic evaluation.

In conclusions, we would like to emphasize the need to consider also PS, besides lung neoplasms and other possible causes, in the differential diagnosis in advanced age patients with pulmonary mass lesions, even if PS is rarely seen. To our knowledge, among the published cases, our bilateral PS is the oldest and the second case over 50 years of age at the diagnosis.

## Consent

Written informed consent was obtained from the patient for publication of this case report and any associated images. A copy of the written consents is available for review by the Editor-in-Chief of this journal.

## Competing interests

The authors declare that they have no competing interests.

## Authors’ contributions

ESE: Conception and design, analysis and interpretation of data, drafting the manuscript and revising it critically for important intellectual content, approving the final manuscript; HB: Conception and design, analysis and interpretation of data, approving the final manuscript; HB: Analysis and interpretation of data, approving the final manuscript; TY: Analysis and interpretation of data, approving the final manuscript; EB: Analysis and interpretation of data, approving the final manuscript.
